# Nurse-patient communication strategies: A proposal of an educational video for Nursing students*

**DOI:** 10.1590/1518-8345.6177.3858

**Published:** 2023-04-17

**Authors:** Jéssica França Pereira, Natália Chantal Magalhães da Silva, Rodrigo Soares Sampaio, Vanessa dos Santos Ribeiro, Emília Campos de Carvalho

**Affiliations:** 1 Universidade Federal do Estado do Rio de Janeiro, Escola de Enfermagem Alfredo Pinto, Rio de Janeiro, RJ, Brasil.; 2 Universidade do Estado do Rio de Janeiro, Hospital Universitário Pedro Ernesto, Rio de Janeiro, RJ, Brasil.; 3 Universidade de São Paulo, Escola de Enfermagem de Ribeirão Preto, Centro Colaborador de la OPS/OMS para el Desarrollo de la Investigación en Enfermería, Ribeirão Preto, SP, Brasil.; 4 Becaria de la Coordenação de Aperfeiçoamento de Pessoal de Nível Superior (CAPES), Brasil.

**Keywords:** Health Communication, Nurse-Patient Relations, Instructional Film and Video, Education, Nursing, Nursing Methodology Research, Nursing, Comunicación en Salud, Relaciones Enfermero-Paciente, Película y Video Educativo, Educación en Enfermería, Investigación Metodológica en Enfermería, Enfermería, Comunicação em Saúde, Relações Enfermeiro-Paciente, Filme e Vídeo Educativo, Educação em Enfermagem, Pesquisa Metodológica em Enfermagem, Enfermagem

## Abstract

**Objective:**

to create, validate and evaluate an educational video on nurse-patient communication strategies for undergraduate Nursing students.

**Method:**

this is a methodological study with a longitudinal design and quantitative analysis. The following stages were conducted: pre-production, production, post-production and evaluation of the video by the target population.

**Results:**

five female nurses evaluated the video storyboard and indicated understanding of the subject matter, the topics addressed and the language used as adequate and pertinent to the theme. Another five female nurses considered the following as present and desirable elements: quality of the audiovisual technique employed, simulated environment, characterization of the characters, and development of the nurse-patient communication strategies The final version of the video was evaluated by nine Nursing students that presented a level of item understanding of at least 96%. The video presents the following strategies: General communication strategies, Intercultural Communication, NURSE, Tell me more, Ask-Tell-Ask, Therapeutic Communication and Communicating Bad News.

**Conclusion:**

this study portrays the creation of a video, its validation by experts and its evaluation by the target population, which indicated it as a relevant educational resource for the teaching-learning process regarding communication strategies. Both the evaluators and the target population considered that the video is a valid instrument to teach content about the nurse-patient communication strategies.

## Abstract (es)

**Objetivo:**

elaborar, validar y evaluar un video educativo sobre estrategias de comunicación enfermero-paciente para estudiantes de la carrera de enfermería.

**Método:**

se trata de un estudio metodológico, con diseño longitudinal y análisis cuantitativo. El público objetivo cumplió las etapas de preproducción, producción, postproducción y evaluación del video.

**Resultados:**

cinco enfermeras evaluaron el *storyboard* del video y consideraron que la comprensión del tema, los tópicos abordados y el lenguaje utilizado eran apropiados y relevantes para el tema. Otras cinco enfermeras consideraron que la calidad de la técnica audiovisual utilizada, el ambiente simulado, la caracterización de los personajes y el desarrollo de las estrategias de comunicación enfermero-paciente estaban presentes y eran adecuados. La versión final del video fue evaluada por nueve estudiantes de enfermería que tuvieron un nivel de comprensión de los ítems igual o superior al 96%. El video presenta las siguientes estrategias: Estrategias Generales de Comunicación, Comunicación Intercultural, *NURSE, Tell me more, Ask-Tell-Ask*, Comunicación Terapéutica y Comunicación de Malas Noticias.

**Conclusión:**

este estudio describe la creación de un video, la validación que realizaron del mismo los expertos y la evaluación del público objetivo, quienes consideraron que es un recurso educativo importante para el proceso de enseñanza-aprendizaje de las estrategias de comunicación. Los jueces y el público objetivo consideraron que el video era un instrumento válido para enseñar las estrategias de comunicación enfermero-paciente.

## Abstract (pt)

**Objetivo:**

construir, validar e avaliar um vídeo educativo sobre estratégias de comunicação enfermeiro-paciente para estudantes da graduação em enfermagem.

**Método:**

trata-se de um estudo metodológico, com delineamento longitudinal e análise quantitativa. Foram percorridas as etapas de pré-produção, produção, pós-produção e avaliação do vídeo pelo público-alvo.

**Resultados:**

cinco enfermeiras avaliaram o *storyboard* do vídeo e apontaram a compreensão do tema, os tópicos abordados e a linguagem utilizada como adequadas e pertinentes à temática. Outras cinco enfermeiras consideraram presentes e desejáveis: qualidade da técnica audiovisual empregada, ambiente simulado, caracterização das personagens e desenvolvimento das estratégias de comunicação enfermeiro-paciente. A versão final do vídeo foi avaliada por nove estudantes de enfermagem que apresentaram nível de compreensão dos itens igual ou acima de 96%. O vídeo apresenta as seguintes estratégias: Estratégias gerais de comunicação, Comunicação Intercultural, NURSE, *Tell me more*, *Ask-Tell-Ask*, Comunicação Terapêutica e Comunicação de Más Notícias.

**Conclusão:**

este estudo retrata a criação de um vídeo, sua validação por peritos e sua avaliação pelo público-alvo, que manifestaram tratar-se de um recurso educativo relevante para o processo de ensino-aprendizagem de estratégias de comunicação. Os juízes e o público alvo consideraram o vídeo como um instrumento válido para ensinar sobre as estratégias de comunicação enfermeiro-paciente.

**Highlights:**

**(1)** Understanding of the subject matter, the language used and the topics addressed were adequate.

**(2)** There was agreement regarding quality, environment, characterization and communication strategies.

**(3)** The final version of the educational video lasts 13 minutes 52 seconds.

**(4)** Nursing students presented an understanding level of at least 96%.

**(5)** It can favor the teaching-learning process and the communication skills.

## Introduction

Considered as the basis of human relations, communication is characterized as a complex process of exchanging or transmitting diverse information, data, emotions and meanings through the use of symbols by means of language, facial expressions, gestures and body postures, between two or more people, and with a purpose^(1-4)^.

In the Nursing context, communication is considered as a basic component for care, as it allows expressing emotions, needs, fears and opinions. Thus, it is considered an important indicator of the quality of the assistance provided, characterizing itself as a key component to implement patient safety^(5-7)^. It is through communication that nurses establish rapport with the multiprofessional team, with the family members and with the patients^(8)^.

With regard to nurse-patient communication, the professionals are the active agents in the communicative process, as they use communicative skills to obtain and provide diverse information about clinical conditions, inform procedures, identify the patient’s needs, promote listening to demands and establish a team-patient-family bond^(3,8)^.

However, for communication to be efficient, it is necessary to overcome certain barriers. They can be related to the interlocutors, to the environment, to the situation, and to the professionals’ skills or expertise. When the barriers are related to the interlocutors, they can present themselves as physical conditions that involve difficulties expressing, receiving or understanding messages, level of hierarchy, forms of approaches or social treatments, use of technical language and cultural differences. Those related to the environment emerge as interferences related to the noise of equipment alarms, precarious lighting, parallel conversation in the environment and privacy; whereas those related to the situation have to do with the relevance given to the subject matter, in terms of communication. In addition, the barriers related to the professionals’ skills and expertise and to empathy are related to the rules and sequences inherent to a conversation^(4,9-10)^.

These interferences can lead to lack of information sharing among team professionals, inconsistencies in medical records, losses in the identification and monitoring of the patient’s clinical signs, and compromise in achieving good results^(10)^. In addition to that, the professionals’ and patients’ safety can also be affected, as failures in communication are pointed out as one of the main causes of adverse events in the Nursing area^(11)^.

The National Curriculum Guidelines for the Nursing course determine the content to be taught and emphasize that communicative skills are competences to be acquired during Nursing training^(12)^. However, some studies point out that communication is insufficiently addressed during undergraduate Nursing studies and that, when it takes place, it is not always able to develop skills that allow the students to deal with the patients’ emotions^(13-16)^.

Thus, given the need to propose forms of innovative strategies and effective methodologies in the teaching of communication, the development, for the time being, of an educational video on communicative strategies in Nursing is justified. It is estimated that using the video in a later study may be able to measure its impact on knowledge acquisition in the area.

For such purpose, the objective of this study was to create, validate and evaluate an educational video on nurse-patient communication strategies for undergraduate Nursing students.

## Method

### Study design, locus and period

This is a methodological study with a longitudinal design and quantitative analysis^(17)^. It was conducted at a public Higher Education Institution in the city of Rio de Janeiro (RJ, Brazil). The process to create the video, validate it with experts and evaluate it with the target population lasted 24 months, encompassing the period from January 2020 to December 2021.

### Participants

According to the literature, there is still no consensus on the number of evaluators required for content evaluation. Some authors indicate that a minimum of five and a maximum of 10 evaluators should participate in the validation process; whereas others suggest from six to 20 participants, with a minimum of three in each group of professionals selected^(18-19)^. A minimum of five evaluators was selected in this paper.

In order to select the participants for validation of the storyboard, the inclusion criteria were the following: experience in the area of nurse-patient communication. In turn, to select the evaluators for video validation, the professionals selected were those with experience in educational and audiovisual resources. The exclusion criterion corresponded to professionals that were not nurses. The following individuals participated in this study: ten nurses, five with experience in the area of communication in Nursing and five with experience in educational resources in health; as well as nine students attending the Nursing course (target population), regularly enrolled in the educational institution where the study took place.

The participants were invited by means of a letter sent to the professionals’ email addresses. All the participants invited accepted to take part in the research.

In relation to the invitation for the target population, the following inclusion criteria were considered: students enrolled in an undergraduate Nursing course from the fourth period onwards and aged more than 18 years old. A total of 25 participants were invited, although only 9 accepted to take part in the research.

### Data collection

Data collection, as well as the instruments used, varied depending on the process to create, validate and evaluate the educational video.

Creation and validation of an educational video comprises three phases: pre-production (writing of the script based on the literature and on the authors’ clinical experience, elaboration and validation of the storyboard by specialists), production (rehearsal with the actors, filming of the scenes, development of images and animations and voiceover), and post-production (editing and validation of the video by people with experience in the area)^(20)^.

During pre-production, the video script and storyboard were prepared^(20)^. In order to write the script, a scoping review on the nurse-patient communication strategies was conducted, with its protocol registered in the Open Science Framework (OSF) under DOI: 10.17605/OSF.IO/26QMX, based on the methodology proposed by the Joanna Briggs Institute (JBI)^(21)^.

Scoping reviews seek to map fundamental concepts of a given area, indicating the main available evidence. It consists of the following stages: identification of the research question, identification of relevant studies, selection of studies and mapping of the findings^(22)^; and presents its protocol based on the PCC strategy (Population, Concept and Context/Scenario)^(23)^. In this research, the Population consisted in the nurse and the patient; the Concept corresponded to the communication strategies; and the Context/Scenario was Nursing.

A primary search was initially conducted in the following databases: Medical Literature Analysis and Retrieval System Online (MEDLINE/PubMed), Cumulative Index to Nursing and Allied Health Literature (CINAHL) and *Literatura Latino-Americana e do Caribe em Ciências da Saúde* (LILACS). Once non- existence of reviews on nurse-patient communication strategies was verified, a detailed search was conducted based on the following strategies: MEDLINE *(“health communication” [MeSH Terms] AND (“nursing” [MeSH Terms] OR “nursing care” [MeSH Terms]))*; CINAHL *(MH communication AND MH nursing care)*; and LILACS *(“comunicação em saúde” [Descritor de assunto] AND “enfermagem” OR “cuidados de enfermagem” [Descritor de assunto])*. Consequently, during the first half of 2020, two independent female researchers sought to answer the guiding question: “Which is the available scientific evidence on the nurse-patient communication strategies?”. The following were considered as eligibility criteria: studies that had nurses and/or patients as participants; that addressed the nurse- patient communication strategies; carried out in the Nursing context/scenario; and with qualitative, quantitative, reflective, descriptive, observational, methodological and review methodologies. Duplicate studies were excluded.

Thus, after extracting the information and based on the experience of the researchers involved, the findings were compiled and categorized to write the script.

The video storyboard was prepared with the purpose of guiding and clarifying the process to create the following stages. It was developed as a chart, with a description of each audiovisual resource to be used, such as the following: scenes, animations, voiceover and background sounds^(20)^.

Once finished, the storyboard was forwarded to be analyzed by five female nurses with experience in the area of communication in Nursing. The instrument for its evaluation was elaborated according to the criteria suggested in a previous study^(24)^, which presented the stages of creation and validation of an educational video on communication in the context of communication in intravenous therapy installation. Thus, the instrument used in this research was based on the judgment of understanding the topic, verbal language adopted and the pertinence of including topics related to the theme (concept of communication, applicability of communication in Nursing, importance of communication in Nursing care, general aspects of nurse-patient communication and nurse-patient communication strategies: Intercultural Communication Strategies; Naming, Understanding, Respecting, Supporting and Exploring (NURSE); Tell me more; Ask-Tell-Ask; Therapeutic Communication: Expression, Clarification and Validation; and Communicating Bad News).

The production stage consists in implementing the ideas elaborated during pre-production^(20)^. Thus, after rehearsing with the actors and making the necessary adjustments to the scenes, the video was recorded in a simulation laboratory at the educational institution, which had similar conditions to the Nursing practice scenario and favorable acoustic conditions.

Subsequently, images and animations were selected, in addition to a presenter for the video.

For the development of images and animations, the legislation referring to the copyright regarding use and reproduction of resources was respected^(25)^. In this way, they were selected from a search in the Google Images tool, provided that they were licensed under the Creative Commons Attribution 4.0 International License and, treated with the Adobe Illustrator and Adobe Flash programs to create images and animations, respectively.

The post-production stage consists in editing the scenes recorded and in validating the video^(20)^. Subsequently, after implementing the necessary adjustments, the video was forwarded to nurses with experience in educational health resources. The instrument for evaluation consisted in judging whether the items – quality of the audiovisual technique employed, the simulated environment, characterization of the characters and development of the nurse-patient communication strategies – were present and desirable. It is noted that, when pertinent, the specialists’ suggestions were accepted by the researchers.

Subsequently, in order to verify understanding and scope of the video content, nine Nursing students expressed their opinions about it.

The students were invited to participate in the study at a predefined moment, after the academic activities, through the Google Meet online platform. After presenting the study objectives and the invitation to participate in the research, those that showed interest in taking part received the link to the Google Forms online form. The evaluation considered an analysis of the understanding regarding each topic addressed, the number of times that it would be necessary to watch the video in order to acquire information, and the quality of the audiovisual material.

The students’ understanding was evaluated by means of a questionnaire with closed questions by resorting to scale from 0 to 10. For each topic covered in the video, 0 represented “The video presented the topic in a NOT COMPREHENSIVE/COMPLETE way”, and 10 represented “The video presented the topic in an EXTREMELY COMPREHENSIVE/COMPLETE way”.

The instruments used in the data collection process of all three stages were created by the authors of this research; therefore, they were not implemented in other studies or validated.

### Data analysis

The records made by the participants, both nurses and students, were stored in a spreadsheet in Microsoft Office Excel®, online version, for descriptive statistical analysis (absolute frequency, relative frequency, mean and standard deviation).

### Ethical aspects

This proposal is part of the research entitled Intervention Strategies in Nursing Teaching and Assistance: Randomized Clinical Trials, registered in *Plataforma Brasil* under Certificate of Presentation for Ethical Appraisal (*Certificado de Apresentação de Apreciação* Ética, CAAE) No. 25629819.5.0000.5285 and approved under opinion No. 3,764,010.

In order to reduce the risks inherent to collection carried out in a virtual format, the records were deleted from the virtual platforms and shared environments, and the data were stored on a local electronic device, thus ensuring secrecy and confidentiality.

In compliance with the recommendations of the relevant legislation, the people who participated in the video scenes signed the Image Use Authorization Form; the presenter signed the Voice Usage Authorization Form; and the expert nurses and students, the Free and Informed Consent Form.

It is also added that the activities developed by the authors and presenter of the video were conducted voluntarily.

## Results

**Figure 1 -:**
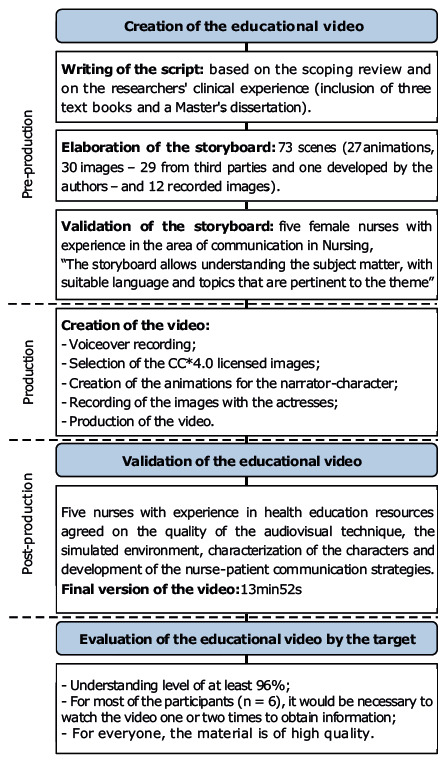
Flowchart corresponding to the process for the creation, validation, and evaluation by the target population of the educational video. Rio de Janeiro, RJ, Brazil, 2022

The process for the creation, validation, and evaluation by the target population of the educational video on nurse-patient communication strategies is presented in Figure 1.

*Creative Commons licenses

### Creation and evaluation of the educational video

**Figure 2 -:**
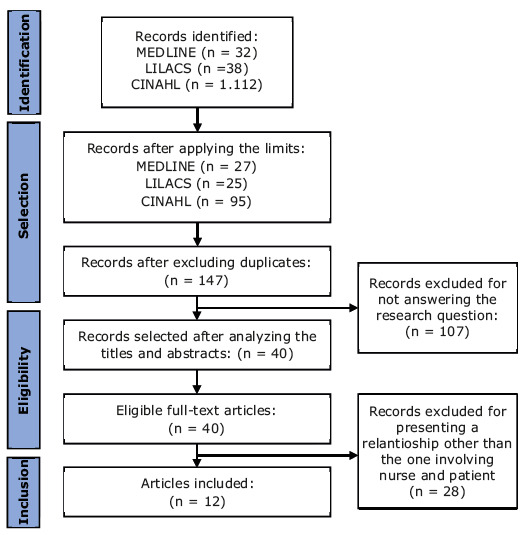
Flowchart corresponding to selection of the articles for the scoping review. Rio de Janeiro, RJ, Brazil, 2022

A scoping review was conducted to develop the script and storyboard. A total of 1,182 studies were identified in the search in the databases. After applying the inclusion criteria, 147 were selected; however, only 12 answered the guiding question and comprised the review, according to the selection flow guided by the extension of the Preferred Reporting Items for Systematic Reviews and Meta-Analyses for Scoping Reviews (PRISMA-ScR) guide^(26)^ shown in Figure 2.

Different nurse-patient communication strategies were identified in this review, namely: Intercultural Communication^(27)^, NURSE, Tell me more and Ask- Tell-Ask^(28)^, Therapeutic Communication^(29)^ and Communicating Bad News^(3,30-31)^. Along with them, a number of studies also presented strategies or techniques for effective communication, without any specific denomination^(28,32-37)^.

Consequently, a category referring to the general aspects of communication was created, being the one most cited across the studies (n = 7). Among other aspects, it addresses actions and recommendations to develop good quality communication^(28,32-37)^. It is noted that, in one study, specific techniques related to surgical procedures were pointed out, which have the purpose of relieving anxiety, fear and anguish, inherent to the pre-, intra- and post-operative periods^(37)^.

With regard to Intercultural Communication, this was mentioned in only one study, defined as the interaction between health professionals and patients from different cultural backgrounds and characterized in three stages: intercultural awareness, intercultural sensitivity and intercultural efficacy^(27)^.

The Communicating Bad News category was cited in two studies and consists in the transmission of unpleasant information related to the patient; information that, in most cases, involves drastic changes in the perception of the future or health prognosis^(3,30-31)^.

On the other hand, Therapeutic Communication is a strategy defined as the professional’s ability to identify the patient’s needs, stimulating their coping and helping them solve their problems, autonomously, as identified by the authors in a study^(29)^.

One of the 11 articles included indicated the NURSE, Tell me more and Ask-Tell-Ask^(28)^ strategies, although not presenting any definition of this concepts; it only describes its functionality and presents examples.

The main information related to the nurse-patient communication strategies identified in the review is arranged in Figure 3.

**Figure 3 -:** Nurse-patient communication strategies identified from the scoping review. Rio de Janeiro, RJ, Brazil, 2022

Nurse-patient communication strategies
General communication aspects^(28,32-37)^: - Search for visual contact and entire focus on the patient; - Clarity and objectivity; - Adaptable to the receiver’s knowledge level and to their needs; - Appreciation of doubts and respect towards the receiver, showing interest and free from judgments; - Guaranteeing a space for the receiver to speak, without interruptions; - Provision of explicit instructions, keeping the patient informed and repeating what was said; - Use of techniques aimed at improving efficiency of the communication process, such as: open questions, reflection statements, and hope and concern statements.
Intercultural Communication^(27)^: - Collection of the maximum information possible about individuals with different cultural characteristics and understanding the essence of - Translation of the content provided to the language spoken by the patient.
*NURSE* * ^(28)^: - Show empathy as a response to the expression of emotions; - Name, understand, respect, offer support and explore the patient’s emotions.
*Tell me more* ^(28)^: - Learning about another person’s perspectives.
*Ask-Tell-Ask* ^(28)^: - Obtain due permission to present information; - Present information clearly; - Verify understanding or agreement.
Therapeutic Communication^(29)^: - Help the patient discover and solve their problems and conflicts, recognize own limitations, adapt to what cannot be changed and face the challenges.
Communicating Bad News^(3,30-31)^: - Provision of emotional support; - Paying attention to the environment where the receiver is; - Knowing when not to speak, knowing how to listen, resorting to an affectionate touch, establishing visual contact and expressing feelings; - Bad news should be transmitted clearly, gradually and in a detailed manner, with truth and objectivity, without punishments or omissions and without resorting to technical terms, jargons or ambiguities; - Use of protocols such as *SPIKES* ^†^; - Stimulating hope based on reality;

* NURSE = Acronym for Naming, Understanding, Respecting, Supporting and Exploring;

† SPIKES = Acronym for Setting up, Perception, Invitation, Knowledge, Emotions and Strategy and Summary

The video script was prepared based on the review findings, shown in Figure 3, and on the experience of the researchers involved, adding three textbooks^(4,38-39)^ and a master’s dissertation^(40)^ that addressed the theme analyzed.

The storyboard was prepared from the script. This, consisting of 73 scenes, addressed the concept of communication, its applicability in Nursing, its importance in care, general aspects of communication and nurse-patient communication strategies (NURSE, Tell me more, Ask-Tell-ask, Therapeutic Communication and Communicating Bad News). The scenes proposed 27 animations, 30 images (from third parties or developed by the authors) and 12 recorded images. Along with this, the following scenes were added: opening, references used, credits, acknowledgments and technical sheet.

Once finished, the storyboard was forwarded to five female nurses with experience on the theme involving nurse-patient communication strategies. All of them were teachers, with a mean age of 35.8 (±6.0) years old, and mean times of professional training and of professional performance of 12.4 (±5.4) and 11.4 (±6.6) years, respectively. Three were specialists and there were four MSc and five PhDs. Regarding performance time in the communication area, the nurses presented a mean of 4.5 (±2.6) years, with three of them having scientific production in the area.

In the storyboard evaluation, there was total agreement among all the nurses, who pointed out that the content allowed understanding the topic and that the language used was easy to assimilate, being adequate to the target audience. In addition, all of them revealed that the topics addressed were pertinent to the theme; however, one indicated that the video did not follow a logical sequence. Thus, the researchers met and, in consensus, implemented the necessary adjustments, accepting those suggestions that they deemed pertinent. The changes made based on the experts’ requests were the following: inclusion of images that represented other means of communication (such as blackboard and gestures); adding the name of the creative and production team at the end of the video; and including the purpose of the NURSE strategy and its representation through examples.

After validation of the storyboard, the process to create the video was initiated with the voiceover recording. Subsequently, the images were selected, the video narrator-character animations were created, and the scenes were recorded.

Once finished, the video was forwarded to five female nurses with experience in educational resources in health. All of them were teachers, with a mean age of 42.8 (±10.2) years old, and mean times of professional training and of professional performance of 18.3 (±11.7) and 19.3 (±13.4) years, respectively. All of them had specialization and Master’s courses, one was a PhD student, and there were four PhDs. Regarding performance time in the area of educational resources in health, the nurses had a mean of 12.3 (±8.4) years and, in addition, a mean of 5.5 (±2.2) years in the area of active methodologies. All had scientific production in the area.

In validating the video, all five nurses considered all the items evaluated as present and desirable, namely: quality of the audiovisual technique used, the simulated environment, characterization of the characters and development of the nurse-patient communication strategies, the video obtained the maximum positive evaluation score.

Table 1 presents the nurses’ evaluation in terms of the topics related to the video components and to the nurse-patient communication strategies.

**Table 1 -:** The nurses’ evaluation (n = 5) in terms of the topics related to the video components and to the nurse-patient communication strategies. Rio de Janeiro, RJ, Brazil, 2022

Topics	Present (n = 5)	Desirable (n = 5)
**Audiovisual technique**		
Initial identification of the content that is intended to be shown	5	5
Lighting required to adequately watch the scenes	5	5
Sound required to hear the narrator’s voice	5	5
When desired, it allows going back to any part of the scenes	5	5
**Environment**		
The scenario reflects the Nursing care practice routine	5	5
The audiovisual material contemplates all the resources required to develop what was presented	5	5
Simplification of the images, animations and scenes does not interfere in the fidelity of what it is intended to be shown	5	5
**Characters**		
The language used corresponds to the one employed in the Nursing practice	5	5
The presenter’s voice is clear	5	5
The tone of the presenter’s voice is adequate	5	5
**Nurse-patient communication strategies**		
The concept of communication	5	5
Applicability of communication in Nursing	5	5
Importance of communication in Nursing care	5	5
General aspects of the nurse-patient communication	5	5
**Nurse-patient communication strategies**		
Intercultural Communication	5	5
NURSE^*^	5	5
Tell me more	5	5
Ask-Tell-Ask	5	5
Therapeutic Communication: Expression	5	5
Therapeutic Communication: Clarification	5	5
Therapeutic Communication: Validation	5	5
Communicating bad news	5	5

* NURSE = Acronym for Naming, Understanding, Respecting, Supporting and Exploring

Given the nurses’ consensus, it can be considered that the audiovisual technique, the environment, the characters and the nurse-patient communication strategies proved to be adequate and appropriate.

The final version of the video lasted 13 minutes and 52 seconds.

The video presented the concepts of the nurse-patient communication strategies found in the literature review by means of playful narration. In the video, the narrator-character (a male nurse) presents the concepts of each strategy speaking directly to the interlocutor. Subsequently, examples of situations are presented through animations in which the nurse employs the previously explained strategy. All the examples mentioned in the video resort to the hospital setting.

### Evaluation of the educational video by the target population

Understanding and scope of the content addressed were evaluated by nine undergraduate Nursing students. Six of them were female, with a mean age of 27.4 (±6.3) years old. It becomes important to note that five were attending the 4^th^ period, two were in the 4^th^ period and another two were in the 3^rd^ period of the course.

Table 2 presents the understanding level presented by the Nursing students in relation to the topics of the video.

**Table 2 -:** Understanding level presented by the Nursing students (n = 9) in relation to the topics of the video. Rio de Janeiro, RJ, Brazil, 2022

Topics	Understanding level (%)
The concept of communication	100
Applicability of communication in Nursing	100
Importance of communication in Nursing care	100
General aspects of the nurse-patient communication	100
Intercultural Communication strategy	97
NURSE^*^ strategy	98
Tell me more strategy	100
Ask-Tell-Ask strategy	100
Therapeutic Communication strategies: Expression	97
Therapeutic Communication strategies: Clarification	100
Therapeutic Communication strategies: Validation	98
Communicating Bad News strategy	98

* NURSE = Acronym for Naming, Understanding, Respecting, Supporting and Exploring

In relation to the number of times that it would be necessary to watch the video in order to acquire information, six students indicated one or two times; and three of them indicated from three to four times. Regarding quality, all of them stated that the material was of extremely high quality.

## Discussion

When performed effectively, nurse-patient communication brings about direct benefits to care, as it favors bonding between the professional and the individual assisted^(1,8)^.

However, a number of research studies point to flaws in this process. The results obtained by a study^(41)^ highlighted the need to teach nurse-patient communication strategies in training programs and qualification courses, in addition to the approach during professional training. Other authors point to the need for a discussion about the techniques that involve such communication strategies^(16,41-43)^.

A research study^(43)^ highlighted the importance of the professionals showing mastery over communication, as this can assist in capturing valuable information and facing challenges. In this way, it is fundamental that the professionals have certain human skills, with communication being the major link in these relationships.

For the development of communicative skills, it is recommended to use different teaching strategies that stimulate learning, considering that a single strategy may not be able to provide all the necessary tools for skills development. Thus, it is emphasized that, when surrounded by active teaching methodologies, the content approach favors knowledge acquisition in a pleasant way^(14)^. In this sense, a study carried out with Nursing professors shows that the development of oral expression, non-verbal communication and listening in Nursing students is favored by active teaching methodologies, such as problem-based learning, problematization and simulation experiences in healthcare settings^(41,44)^.

That said, using videos as a teaching-learning strategy is considered promising for the development of knowledge involving professional training^(45)^. Therefore, the proposal to create, validate and evaluate an educational video focused on nurse-patient communication strategies can favor the development of skills of nurses in training.

One study showed that students had greater knowledge acquisition in the area of peripheral venipuncture after using a validated educational video on the theme^(30)^. In addition to that, using educational videos in student training can help fix technical knowledge and enable greater preparation and increased confidence in the performance of procedures^(46)^.

Among other topics, the video presents general aspects of nurse-patient communication, the Intercultural Communication Strategy, the *NURSE* Strategy, the Tell me more Strategy, the Ask-Tell-Ask Strategy, the Therapeutic Communication Strategies: Expression, Clarification and Validation; and the Communicating Bad News Strategy.

It is understood that the general aspects of nurse-patient communication are related to communication techniques involving nurse-patient that do not receive a specific nomenclature; however, they can be used in most communicative processes, as they enable effective communication, easing information sending and reception, generating bonding, trust and support for the patient^(28,32-37)^.

With regard to the Intercultural Communication Strategy, it aims at facilitating egalitarian dialogue between nurse and patient, so that the differences related to the ethnic and cultural diversities of those involved are respected^(47)^. Lack of understanding about the patient’s representations and beliefs generates ethnocentric care, which can compromise adherence to treatment and, consequently, the results achieved^(48)^.

Regarding the NURSE Strategy, acronym for Naming, Understanding, Respecting, Supporting and Exploring, the objective is to understand and accept the patient’s emotions. To apply it, the professional should do the following: name the perceived emotions, show understanding of the patient’s feelings, show respect for the emotions expressed by the patient, provide the necessary support and encourage coping, and demonstrate interest in what concerns the patient^(4,16,49)^.

The Tell me more Strategy allows the professional to understand the patient’s emotions; there is a stimulus for verbalization^(16)^. For the professional to be able to employ it, it becomes necessary to recognize that communication will be based on three levels: understanding the information, understanding how the patient deals emotionally with the content conveyed, and understanding the meaning of the information for the patient^(4)^.

Similarly, the Ask-Tell-Ask Strategy is divided into three stages and aims at strengthening the nurse-patient bond^(16)^. In the first stage, Ask, the professional evaluates the patient’s doubts and questions; in the second, Tell, the nurse clearly answers the information that needs to be transmitted and; in the third and last step, Ask, the patient’s understanding of the information received is verified^(4,16,49)^.

Regarding the Therapeutic Communication Strategies: Expression, Clarification and Validation, the focus is on helping the patient to deal with their problems, recognize own limits, adapt to the new reality and face the challenges. This strategy is divided into three groups: Expression, Clarification and Validation. In Expression, the professional encourages the patient to verbally express their thoughts and feelings, being more employed in the initial communication stage; in Clarification, the nurse tries to understand the message sent by the patient, with the possibility of requesting comparisons and descriptions in a logical sequence and; lastly, Validation seeks to ensure that the messages transmitted have been understood, and repetition of what was informed may be requested^(4,16)^.

The Communicating Bad News Strategy is understood as the transmission of unpleasant information, such as negative prognoses, which lead to drastic changes in the patient’s lifestyle^(3)^. In this case, the focus needs to be on clarity, objectivity, honesty, detail and absence of omissions, having emotional support as a sustaining element^(3,31)^. In order to support transmission of this information, there are guides and protocols such as SPIKES, acronym for Setting up (preparing for the meeting), Perception (perceiving the patient), Invitation (inviting for dialogue), Knowledge (transmitting the information), Emotions (expressing emotions) and Strategy and Summary (summarizing and organizing strategies)^(16,50)^. This has the following objectives: understanding how the patient and their family members understood the message sent, providing information according to what the patient wants/bears receiving at the moment, welcoming the reactions to the content conveyed, and establishing a care plan^(51)^.

In relation to the video length, the final version lasted 13 minutes and 52 seconds, within the maximum limit recommended in studies involving the development of educational resources, which would be 15 minutes. Exceeding this time can make the experience of watching the video tiring and cause dispersion in the viewers^(46,52)^.

The steps used to create and validate videos proved to be adequate to obtain a final product that is accurate, reinforcing their use in research instruments on this theme. An example is using video as a trigger for a situation that involves the teaching-learning process, with regard to nurse-patient communication for undergraduate Nursing teaching^(53)^.

The contributions of the video produced from this study to the advancement of scientific knowledge for Nursing are linked to the provision of a reliable educational resource that can favor the teaching-learning process. This can be considered a tool for the development of communicative skills in Nursing students, thus contributing to improving the care provided.

As limitations of the current research, the small number of evaluators in each stage of the study can be considered, even if there was a high rate of satisfactory answers regarding evaluation of the storyboard, the video and its applicability.

## Conclusion

This study covered the creation process corresponding to an educational video on nurse-patient communication strategies, positively evaluated by nurses with experience in the area regarding quality of the audiovisual technique employed, the simulated environment, characterization of the characters and development of the nurse-patient communication strategies. The material produced was also submitted to evaluation by the target audience, which pointed out a high level of understanding in each topic addressed (> 96%) and the extremely good quality of the audiovisual material (100%).

However, it is recommended to conduct studies that assess changes, which can be attributed to the educational video, in the students’ knowledge acquisition in terms of nurse-patient communication strategies.
